# A conformational change of C-reactive protein drives neutrophil extracellular trap formation in inflammation

**DOI:** 10.1186/s12915-024-02093-8

**Published:** 2025-01-07

**Authors:** Ebru Karasu, Rebecca Halbgebauer, Lena Schütte, Johannes Greven, Felix M. Bläsius, Johannes Zeller, Oscar Winninger, David Braig, David Alexander Christian Messerer, Bettina Berger, Hendrik Feuerstein, Anke Schultze, Karlheinz Peter, Uwe Knippschild, Klemens Horst, Frank Hildebrand, Steffen U. Eisenhardt, Markus Huber-Lang

**Affiliations:** 1 Institute of Clinical and Experimental Trauma Immunology, University Hospital Ulm, Helmholtzstrasse 8/1, 89081 Ulm, Germany; 2 Department of Orthopedic Trauma Surgery, RWTH Aachen University, Aachen, Germany; 3 Department of Plastic and Hand Surgery, Medical Faculty of the University of Freiburg, University of Freiburg Medical Centre Freiburg, Freiburg, Germany; 4 Baker Department of Cardiometabolic Health, University of Melbourne, Melbourne, Australia; 5 Baker Heart and Diabetes Institute, Melbourne, Australia; 6 Department of General and Visceral Surgery, Ulm University Medical Center, 89081 Ulm, Germany

**Keywords:** C-reactive-protein (CRP), Monomeric CRP (mCRP), Neutrophil activation, Neutrophil extracellular trap (NET), Inflammation, Severe injury, Polytrauma (PT)

## Abstract

**Background:**

C-reactive protein (CRP) represents a routine diagnostic marker of inflammation. Dissociation of native pentameric CRP (pCRP) into the monomeric structure (mCRP) liberates proinflammatory features, presumably contributing to excessive immune cell activation via unknown molecular mechanisms.

**Results:**

In a multi-translational study of systemic inflammation, we found a time- and inflammation-dependent pCRP dissociation into mCRP. We were able to confirm that mCRP co-localizes with leukocytes at the site of injury after polytrauma and therefore assessed whether the CRP conformation potentiates neutrophil activation. We found mCRP-induced neutrophil-extracellular trap formation in vitro and ex vivo involving nicotinamide adenine dinucleotide phosphate oxidase activation, p38/mitogen-activated protein kinase signaling, and histone H3 citrullination. Mimicking the trauma milieu in a human ex vivo whole blood model, we found significant mCRP generation as well as NET formation, prevented by blocking pCRP conformational changes.

**Conclusions:**

Our data provide novel molecular insights how CRP dissociation contributes to neutrophil activation as driver of various inflammatory disorders.

**Supplementary Information:**

The online version contains supplementary material available at 10.1186/s12915-024-02093-8.

## Background

Native C-reactive protein (CRP) circulates as a pentamer (pCRP) composed of five identical and non-covalently bound subunits. There is strong evidence that on biological surfaces, pCRP dissociates into an intermediate state (termed pCRP*), exposing neo-epitopes, losing the non-covalent bonds and exerting major proinflammatory effects in vivo [1, 2]*.* pCRP* can further dissolve into its monomeric subunits (mCRP), which share the same bioactive neo-epitopes as well as inflammatory properties as pCRP* [3]. Since pCRP* cannot be purified, mCRP can be used as surrogate to study pCRP* effects in vitro [3]. As shown in several experimental models of inflammation, mCRP is associated with the exacerbation of inflammation, whereas immune-regulatory functions appear to be attributed to native pCRP. In vivo clinical as well as animal studies demonstrated that mCRP increases during infection, thrombo-inflammatory and cardiovascular events [3–6]. We previously reported in a rat model of ischemic reperfusion injury that conformational changes of CRP exert proinflammatory effects via interaction with leukocytes [7, 8]. However, the underlying molecular mechanisms remain to be elucidated. Neutrophils are key players in the initiation and modulation of immune responses to inflammation and major mediators in trauma-related diseases [9]. DNA shedding, termed as neutrophil extracellular trap (NET), represents a unique antimicrobial strategy. However, in several inflammatory conditions, including multiple injury, the release of NETs accompanied by mediators including myeloperoxidase (MPO), neutrophil elastase, and histones, is associated with the exacerbation of inflammation and organ injury, culminating in (multiple) organ failure and increased mortality [10, 11].

Therefore, we examined the role of the CRP conformation in neutrophil activation in systemic inflammation. For the first time, we provide evidence that pCRP*/mCRP induces NET formation in vitro and ex vivo. Moreover, we tested mechanisms to prevent NET formation by (i) peptidylarginine deiminase 4 (PAD4) inhibition in vitro and (ii) blockade of pCRP dissociation by an established small molecule inhibitor [3, 12, 13] ex vivo under inflammatory conditions. In a translational approach, we investigated the importance of CRP conformation during systemic inflammation in a well-established porcine polytrauma (PT) model as well as in severely injured patients.

## Results

### pCRP is the main conformation in plasma whereas pCRP*/mCRP is deposited at the site of injury

**Fig. 1:**
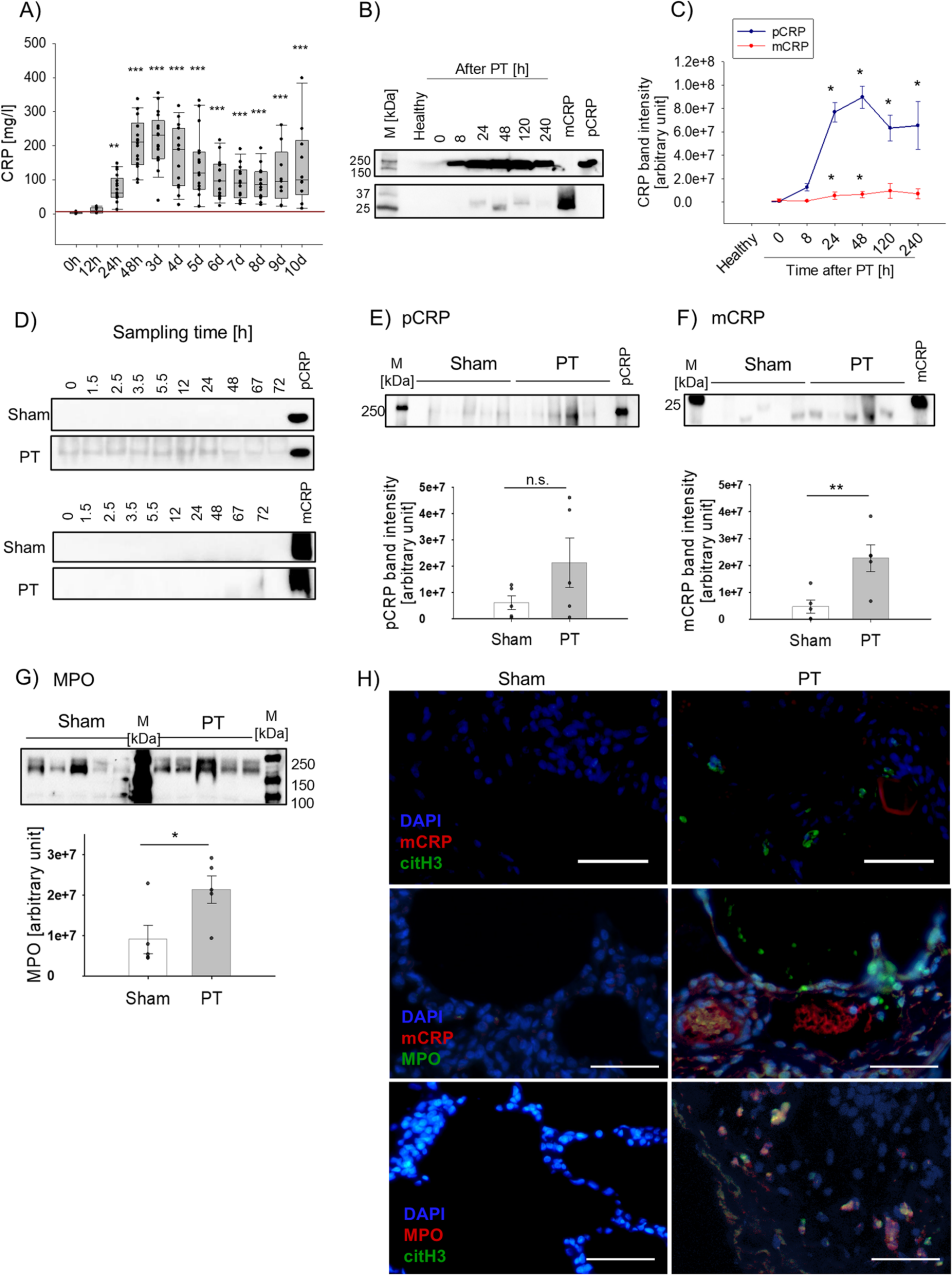
CRP conformation in clinical and experimental PT. **A** Temporal course of plasma CRP levels after huPT represented by vertical box plots, plotting the median, 10th, 25th, 75th, and 90th percentiles with error bars. The red line indicates the physiological concentration of CRP (0—5 mg/l), *n* = 17. One-Way ANOVA followed by Dunn´s method for post-hoc testing. **B** Representative native CRP immunoblot including healthy and huPT plasma samples. **C** Densitometry analysis of pCRP and mCRP band intensities in plasma samples. One-Way ANOVA followed by Dunn´s method post hoc testing (*n* = 7 healthy, *n* = 8–13 huPT). **D** Representative immunoblots of porcine plasma samples obtained from sham and pPT animals at different sampling times and probed for pCRP (upper panel) and mCRP (lower panel). Normalized band intensity of (**E**) pCRP and (**F**) mCRP in porcine lung tissue lysates. *n* = 5, Student´s t-test. **G** Immunoblot probed for myeloperoxidase (MPO) and normalized band intensities in porcine lung tissue homogenates, *n* = 5, Student´s t-test. **H** Representative immunofluorescent image of pig lung sections after mCRP, citrullinated Histone H3 or MPO (lower panel) and nuclei staining (DAPI, blue). Scale bars indicate 50 µm. * *p* < 0.05, ** *p* < 0.01, *** *p* < 0.001

To study CRP conformation, we assessed a human cohort and an animal model of multiple injury which has been characterized in previous studies to induce a strong systemic inflammatory response accompanied by major alterations of innate immune functions [14, 15]. In a prospective, observational, mono-centered study of severely injured patients (human polytrauma, huPT) (Table 1), plasma CRP levels displayed a significant and time-dependent increase ranging from 10–400 mg/L (Fig. 1A) consistent with earlier findings [16, 17]. Moreover, plasma CRP concentrations correlated significantly with leukocyte counts, clinical scores and the hospital length of stay (Additional file 1: Fig. S1A-F). However, several studies reported controversial results on the predictive value of CRP for trauma injury severity and inflammation [18, 19], which was suggested by its slow kinetics compared to other biomarkers [20] as well as its conformation. So far, there is no information about the CRP conformation post trauma since clinical assays are not able to distinguish between pCRP and mCRP. Therefore, we performed native western blot analyses with huPT plasma and found a significant increase in pCRP levels (Fig. 1B). Remarkably, significantly increased mCRP was detected after 8 h, peaking 48 h after PT. Densitometric analysis of pCRP and mCRP band intensities demonstrated that the majority of plasma CRP was present as pCRP (Fig. 1C). In healthy control plasma, neither pCRP nor mCRP could be detected. In a translational effort, we performed a post hoc subgroup analysis in a clinically relevant porcine model of severe multiple injury including lung contusion, liver laceration and hemorrhagic shock [21–23] (porcine polytrauma, pPT). In porcine plasma samples, we observed a clear signal for pCRP, whereas little mCRP was present (Fig. 1D). We further analyzed the lung as the site of injury because (I) acute lung injury and acute respiratory distress syndrome are frequent manifestations of multiple organ failure during inflammation [24, 25] and (II) maladaptive neutrophil functions trigger the release of toxic granular contents and NET formation that are involved in lung inflammation [26]. In the lung tissue of pPT animals, pCRP underwent conformational changes, as indicated by strong pCRP*/mCRP staining in lung sections using conformation-specific mouse anti-human antibodies (pCRP (8D8), mCRP (9C9)), which showed cross-reactivity with porcine CRP (Additional file 1: Fig. S2A and S2B). Densitometric analysis showed significantly more mCRP in pPT lung tissue than in sham animals (Additional file 1: Fig. S2C). Native western blots of lung lysates confirmed significantly increased mCRP after pPT, which was not observed in sham animals (Fig. 1F). Although increased by trend, pCRP was not significantly altered between sham and PT animals (Fig. 1E). Our previous studies indicate that in vitro and in vivo pCRP binding to bioactive lipid rafts, found in inflamed tissues and activated monocytes, represents an essential pathophysiological event leading to pCRP dissociation into neo-epitope exposing CRP [7, 27]. Therefore, we suggest that injured organs expose similar lipids including phospholipids facilitating pCRP dissociation and mCRP deposition. In addition, CRP staining was detected in co-localization with inflammatory cells (Additional file 1: Fig. S2B, lower panel). Moreover, signs of neutrophil activity, reflected by the antimicrobial enzyme MPO, were found in the traumatized lung. Significantly increased local MPO release indicated lung inflammation due to enhanced neutrophil infiltration and activation (Fig. 1G) [25]. The presence of cells positive for MPO and citrullinated H3 (citH3) in proximity to mCRP in lung tissue after pPT pointed to local neutrophil infiltration and NET formation (Fig. 1H).

**Table 1:** Clinical data of study participants

	**huPT (*n* = 17)**	**Healthy (*n* = 7)**
**Demographic data**	Mean ± SEM; Median (min.-max.)	Mean ± SEM; Median (min.-max.)
Age	40.5 ± 3,6; 46 (22–58)	36.1 ± 2.7; 37 (25–47)
Sex, m/f (n)	14/ 3	4/3
**Injury Severity and clinical course**		
ISS	37 ± 2,7; 37 (22–57)	n/a
GCS	3 ± 0; 3(3–3)	
ICU stay (d)	16,2 ± 3,7; 14 (3–42)	n/a
Nosocomial Infection, n (%)	5 (30%)	n/a
Death, n (%)	3 (18%)	n/a
**Mechanism of injury**		
Motor vehicle accident, n (%)	13 (76.4%)	n/a
Falls, n (%)	4 (23.5%)	n/a

*Abbreviations*: *GCS* Glasgow Coma Scale, *ICU* intensive care unit, *ISS* injury severity score

### mCRP induces PAD4-dependent NET formation in neutrophils

**Fig. 2:**
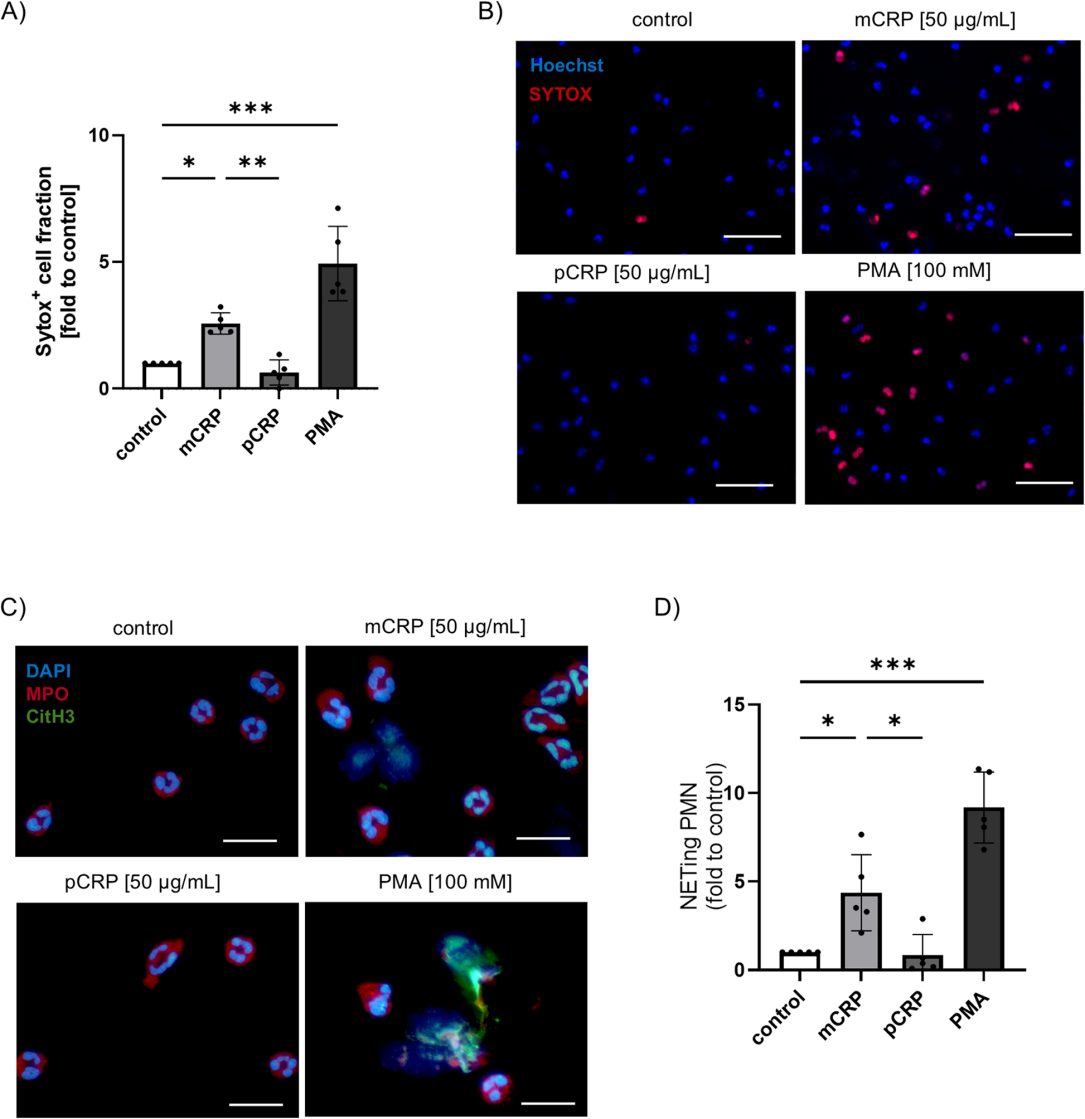
mCRP induces NETosis in human neutrophils in vitro. **A** Freshly isolated neutrophils from health volunteers after treatment with pCRP (50 µg/mL), mCRP (50 µg/mL) and PMA (100 mM) for 1.5 h. DNA was stained using DAPI (blue) and SYTOX™ (red). SYTOX + cell fraction, normalized to control in HBSS + + buffer, after 1.5 h. *n* = 5. **B** Immunofluorescence image of PMN stained with DAPI (blue) and SYTOX.™ (red) after 1.5 h of treatment. Images are representative of 5 different donors. Scale bars indicate 50 µm. **C** PMNs were treated as described above for 1 h; cytospins were prepared, fixed and stained. PMNs show staining for MPO (red), citH3 (green) and nuclei staining (DAPI, blue) as merged channel image. Scale bars indicate 25 µm. **D** The proportion of NETing cells in **C**, i.e. of citH3-positive cells and cells with decondensed chromatin normalized to control samples 1 h after stimulation. *n* = 5

To further elucidate the impact of CRP conformation on neutrophil function, we exposed human neutrophils from healthy donors with 50 µg/mL of either pCRP or mCRP corresponding to plasma concentrations during acute inflammation [16, 17]. PMNs were stained with membrane-permeable Hoechst and membrane-impermeable SYTOX to distinguish between intracellular DNA in intact and necrotic cells as well as decondensed chromatin released into the supernatant medium during NETosis. SYTOX-positive cell fractions after 1.5 h were normalized to non-stimulated controls. Compared to the controls, mCRP-exposed cells demonstrated a significant increase in NET formation shown by a 2.5 higher number of SYTOX positive cells which was increased to a fold change of 5 in the positive control (100 nM PMA), while pCRP did not induce NETosis (Fig. 2A-B). As observed after 2.5 h, SYTOX staining was mainly visible in cells demonstrating decondensed chromatin which occurs at later stages during NET formation (Additional file 1: Fig. S3A).

To further assess the process of NET formation, we stimulated PMNs from healthy donors as described above and subsequently stained them for citH3, MPO, and DAPI. Remarkably, after one hour, we already visualized an increase in citH3-positive cells in mCRP-treated cells compared to controls, suggesting the appearance of early stages of NET formation with a co-occurrence of chromatin and citH3 extrusion (Fig. 2C). Images depicting individual stainings for MPO, citH3, and DAPI are included in Additional file 1: Fig. S3B. Comparing citH3-positive cells to the untreated control revealed significant NET formation by mCRP (Fig. 2D).

**Fig. 3:**
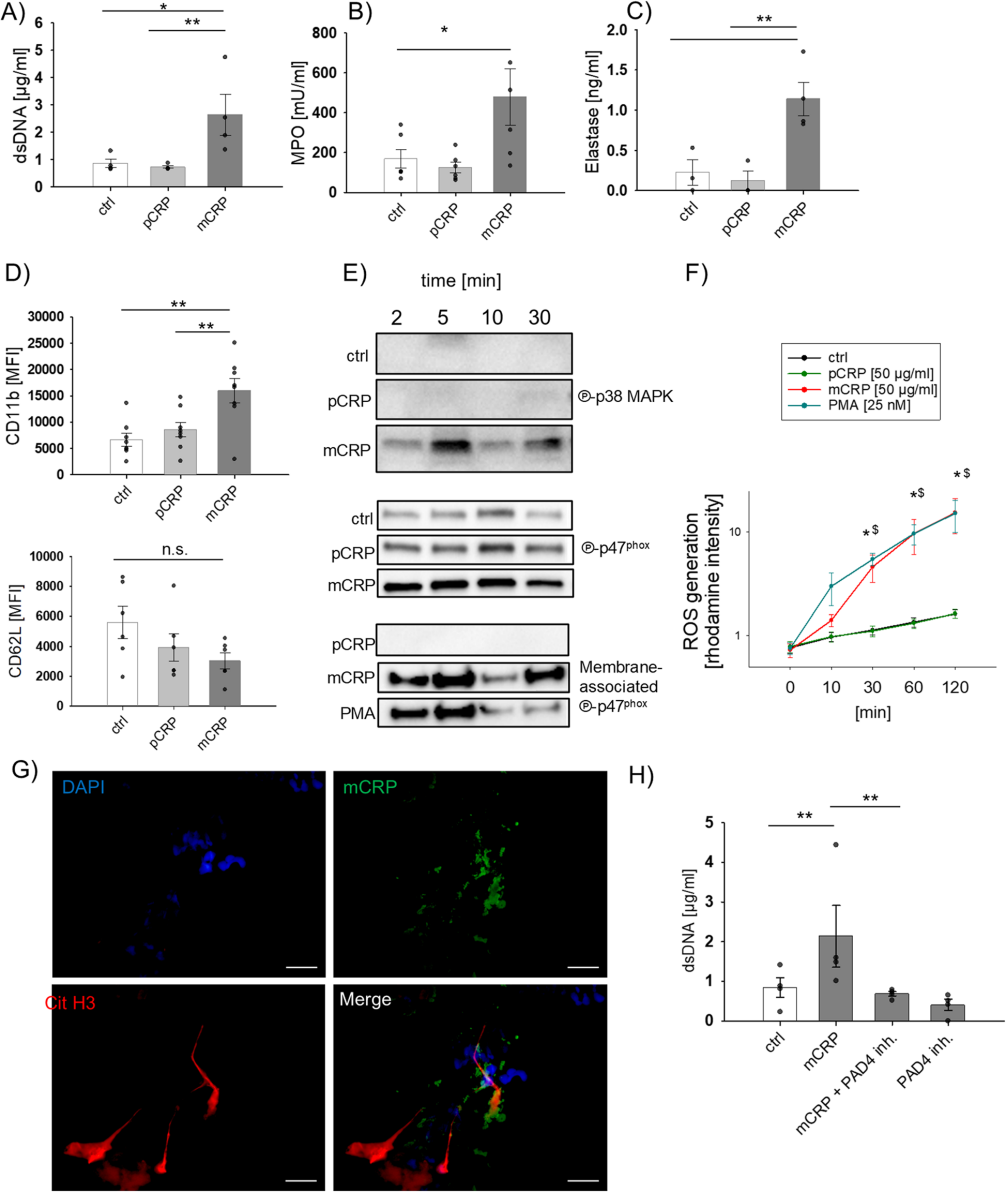
mCRP, but not pCRP activates resting PMNs and induces PAD4-dependent NET formation in vitro. **A** PMN supernatants were assessed for cell-free DNA (dsDNA) *n* = 4 per group, **B** MPO, *n* = 6 per group, and **C** Elastase, *n* = 4 per group. **D** Neutrophil activation was assessed by surface marker expression of CD11b, *n* = 8 and CD62L, *n* = 6. **E** Representative immunoblots for ℗-p38 MAPK (upper panel), and ℗-p47phox (middle) in PMN cell lysates after incubation with pCRP and mCRP. In the lower panel, the amount of translocated ℗-p47phox was assessed in PMN protein membrane fractions. **F** ROS generation was determined by rhodamine intensity in neutrophils after stimulation at indicated time points. PMA served as positive control. *N* = 4. *p < 0.05 for mCRP group vs. ctrl and pCRP treatment groups. $ *p* < 0.05 for PMA group vs. ctrl and pCRP treatment groups. **G** Immunofluorescence image of mCRP-treated PMN cytospins show mCRP (green), citH3 (red) and nuclei staining (DAPI, blue) as single channel and merged channel image, respectively. Image was taken at 630 × magnification. **H** Measurement of the dsDNA content in cell-free PMN supernatants treated with mCRP (50 µg/mL), a PAD4-specific inhibitor (PAD4 inh), and combined mCRP and PAD4 inh. cells, *n* = 4. One-Way ANOVA followed by Student Newman-Keuls post hoc testing was used

To confirm that mCRP induces NET formation, PMNs were incubated with mCRP, stained with DAPI (Additional file 1: Fig. S4A), and established NET markers, including cell-free double-stranded DNA (dsDNA), MPO and elastase [10] were analyzed in the supernatant. As presented in Fig. 3A-C, all of these NET-relevant markers were significantly increased in neutrophil supernatant fluid after mCRP incubation. Studies demonstrated that NET formation follows a well-orchestrated program involving cell activation and upregulation of adhesion markers [28]. In line with this finding, mCRP exposure induced a significant increase in CD11b and tendential decrease in CD62L expression in neutrophils, indicating cell activation (Fig. 3D).

To determine the involved molecular pathways of mCRP-induced NET formation, analysis of different signal molecules including the p38 mitogen-activated protein kinase (MAPK) signaling pathway [29] was assessed. Indeed, mCRP activated the p38 MAPK signaling pathway as reflected by p38 phosphorylation (Fig. 3E, upper panel). Given that NET formation involves oxidative mechanisms including ROS generation by nicotinamide adenine dinucleotide phosphate (NADPH) oxidase activity, we next examined the phosphorylation state of p47^phox^, an essential component of the NADPH oxidase [29]. In this context, we included phorbol-12-myristat-13-acetate (PMA) as positive control since it is known as an oxidative-dependent NET inducer [30]. After exposure to mCRP, p47^phox^ was detected in a phosphorylated state in PMN cell lysates (Fig. 3E, middle panel). Accordingly, membrane fractions obtained from cell lysates displayed a strong signal for phosphorylated p47^phox^, confirming translocation of key components and subsequent activation of NADPH oxidase (Fig. 3E, lower panel). Moreover, reactive oxygen species (ROS) generation as further indicator of NOX activity was observed in PMNs after incubation with mCRP (Fig. 3F). In a recent study, a direct link between ROS generation and PAD4 activity has been described in human neutrophils during NET formation [31]. For this purpose, we performed immunostaining for citrullinated histone H3 (citH3), representing a PAD4-specific histone modification [32]. Indeed, we found positive staining for citH3 in co-localization to mCRP in PMNs after exposure to mCRP. (Fig. 3G). To exclude the possibility that mCRP itself aggregated during incubation and positive staining only reflected aggregated mCRP protein, immunofluorescent staining of mCRP alone in concentrations employed in our assays was performed. No staining was observed (data not shown). In presence of GSK484, as PAD4-specific NET inhibitor [33], mCRP-induced NET formation was significantly reduced as assessed by dsDNA release into the supernatant (Fig. 3H). Our data strongly indicate that mCRP induces a p38-MAPK-, NADPH oxidase, ROS- and PAD4-dependent NET formation in human neutrophils.

Besides NET formation, analysis of additional important neutrophil effector functions revealed that mCRP itself possesses some chemotactic potential and by trend impairs chemotaxis towards bacterial components (*p* = 0.1), while phagocytosis of *Escherichia coli* bioparticles was unchanged (Additional file 1: Fig. S4B-D). By contrast, all these effects were completely absent in the presence of pCRP, highlighting the importance of the CRP conformation. This can also be explained by conformation-specific receptor binding on cells.

### pCRP dissociation is induced by inflammatory conditions and can be inhibited by 1,6-bis(phosphocholine)-hexane (1,6-bis-PC)

**Fig. 4:**
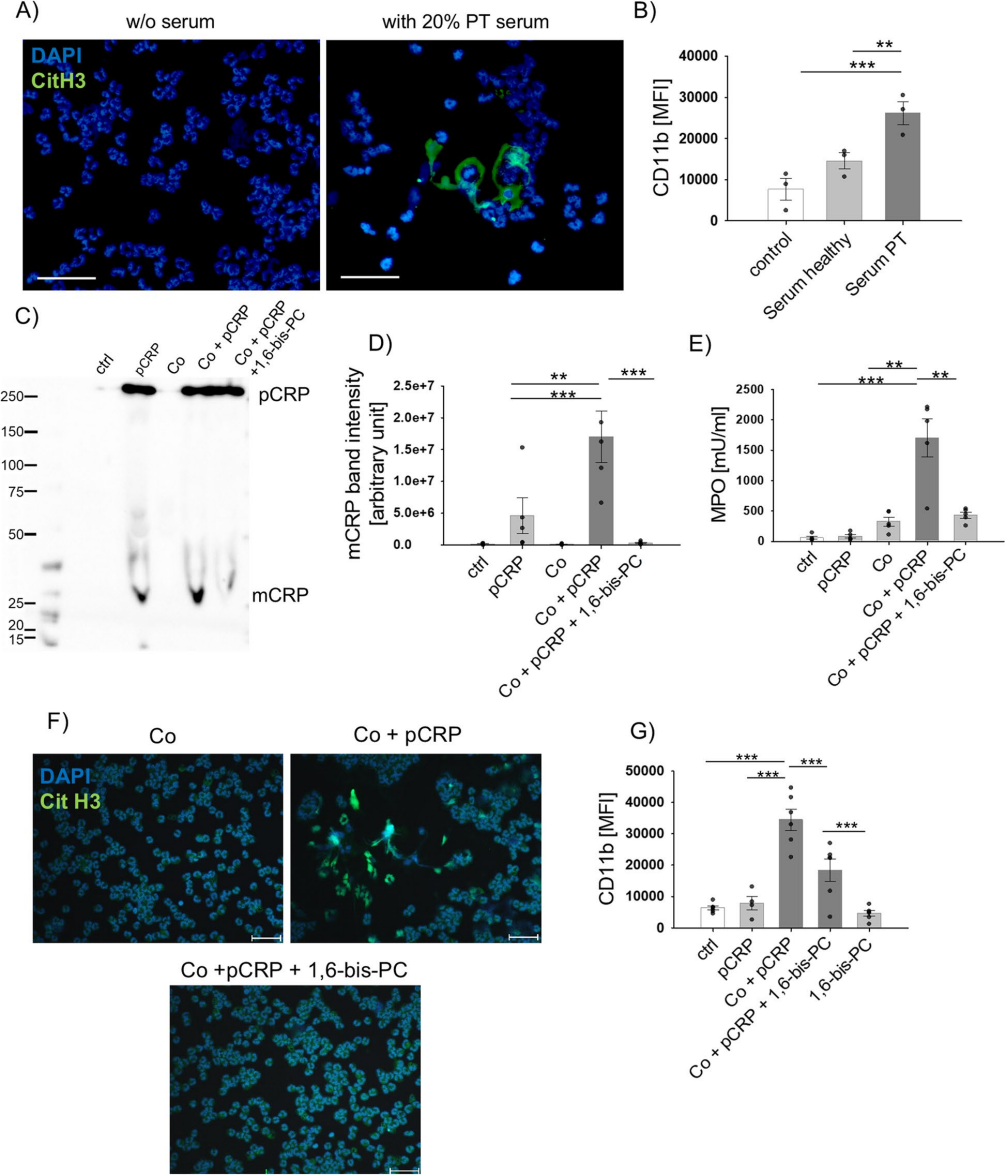
Inflammation-induced pCRP dissociation can be blocked by 1,6-bis-PC which effectively inhibited neutrophil activation and NET formation. **A** Representative immunofluorescence image of citH3 staining in PMNs after 1 h incubation with 20% huPT serum. Green, citH3; blue, DAPI **B** PMN CD11b surface marker expression after incubation with 20% healthy serum or huPT serum. PMNs without serum incubation served as control, *n* = 3. **C** Native western blot image probed for CRP conformation. Human ex vivo whole blood stimulation with a clinically relevant inflammatory cocktail (Co) including C3a (1000 ng/mL), C5a (100 ng/mL), IL-6 (500 pg/mL), IL-8 (150 pg/mL), and IL-1β (200 pg/mL) alone or in combination with pCRP (50 µg/mL), or pCRP + 1,6-bis-PC for 2 h. **D** Densitometric analysis of mCRP band intensity (~ 25 kDa) in plasma samples, *n* = 5 per group. **E** MPO content in plasma samples after ex vivo experiments, *n* = 5 per group. **F** citH3 and nuclei staining in fixed PMNs after 2 h stimulation, *n* = 5 per group, green, citH3; blue, DAPI. **G** CD11b expression on PMNs after stimulation *n* = 5–6 per group. *p < 0.05, **p < 0.01. Scale bars indicate 50 µm

Prompted by our in vivo and in vitro findings that mCRP deposition was present at the injury site and associated with NET formation, we further investigated the involvement of the trauma-relevant inflammatory environment on neutrophil activity. In a translational approach, neutrophils isolated from healthy donors were incubated in 20% human serum from either PT patients or healthy probands. We found DNA spreading, citH3 signal, and neutrophil activation by CD11b, indicating NET formation induced by PT serum (Fig. 4A and B). We hypothesized that neo-epitope exposing CRP in PT plasma was responsible for these effects. Recent literature data described in vitro pCRP dissociation in the presence of lipopolysaccharide and after binding to lipid-rich rafts originating from damaged cells [3, 27]. To gain further insight into the inflammation setting, we investigated whether inflammation leads to CRP dissociation by using an animal-free ex vivo whole blood approach. Human whole blood was incubated with pCRP alone or a combination with a cocktail of key inflammatory mediators (Co), including the complement factors C3a and C5a as well as the interleukins (IL) IL-6, IL-8 and IL-1β. Strikingly, in the presence of Co + pCRP, we found significantly increased mCRP, indicating pCRP dissociation ex vivo (Fig. 4C, D). Moreover, significantly increased MPO release was detected in plasma samples after incubation with Co + pCRP (Fig. 4E). To prevent pCRP dissociation and downstream inflammatory effects, we included 1,6-bis(phosphocholine)-hexane (1,6-bis-PC) [12]. Indeed, 1,6-bis-PC inhibited pCRP dissociation into mCRP (Fig. 4D) and consequent MPO release in whole blood (Fig. 4E). Furthermore, we found signs of NET formation (DNA spreading by DAPI staining and citH3) and changes in CD11b expression induced by the Co-induced pCRP dissociation in isolated neutrophils. Again, pre-incubation with 1,6-bis-PC inhibited these effects, representing a potential therapeutic modulation of CRP dissociation and mCRP-induced inflammatory effects (Fig. 4F-G).

## Discussion

**Fig. 5:**
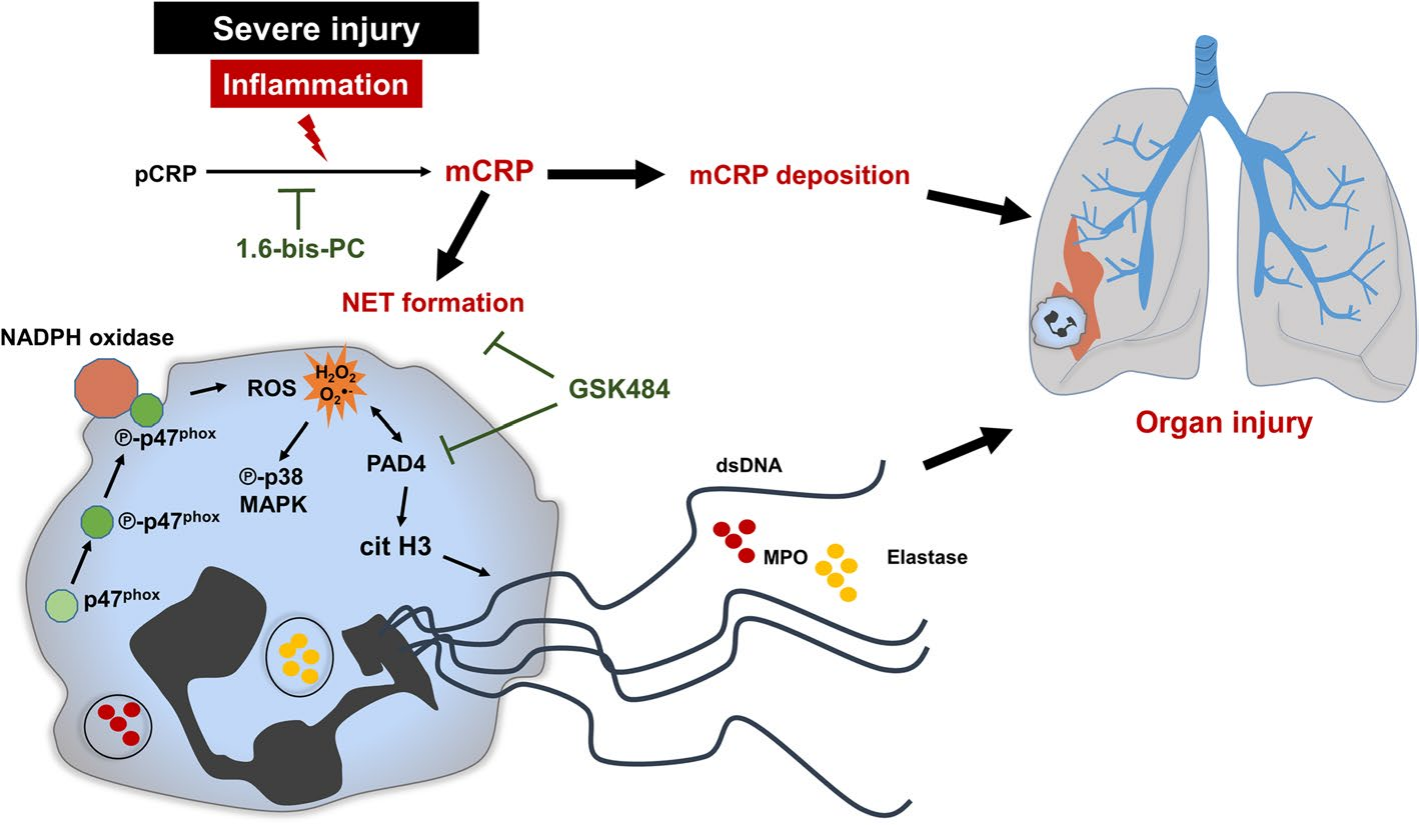
Proposed mechanism of mCRP-driven neutrophil dysfunction. PT triggers a complex inflammatory response leading to pCRP dissociation to mCRP. At the site of injury, mCRP deposition co-localizes with inflammatory cells, which can exacerbate inflammation and can culminate into organ injury. In contrast to pCRP, mCRP alters important neutrophil effector functions. Among them, mCRP initiates activation of neutrophils by increasing CD11b and by decreasing CD62L expression and induces NET formation in neutrophils. Mechanistic pathways involve activation of the p38 MAPK signaling pathway and activation of NADPH oxidase by phosphorylation and translocation of p47^phox^ to the cellular membrane. Moreover, ROS production and histone H3 modification by PAD4 involves the release of dsDNA, MPO, and Elastase, which might contribute to an increased inflammatory microenvironment and organ injury. Blockade of pCRP dissociation inhibits downstream inflammatory responses. Blockade of PAD4 activity by GSK 484 inhibits dsDNA release and thus NET formation

In this study, we provide evidence that a structural change of pCRP into neo-epitope-exposing CRP (pCRP*/mCRP) is responsible for aggravation of inflammation after tissue injury. Mechanistically, we found a strong induction of NET formation by mCRP as summarized in Fig. 5.

CRP blood concentrations, as crucial component of the acute phase response, can rise up to 1000-fold after trauma and sepsis [4, 34]. In line, we found a time-dependent increase of CRP in our PT patient cohort. Using native western blot analysis, we demonstrated the presence of mCRP in huPT plasma, indicative for CRP dissociation. However, this method did not allow us a precise quantification of the amount of the respective CRP conformation due to the non-homogenous band pattern of mCRP under native western blot assay conditions. Therefore, the diagnostic potential of mCRP remains unknown due to the lack of a reliable assay for clinical routine testing. Further studies in a larger PT patient cohort with a more conformation-specific assay method for CRP might help to study correlation between pCRP and mCRP in plasma samples and to evaluate their respective potential as marker for diagnostic purposes. Recently, a redox sensitivity-based method using a single assay was reported to quantify both pentameric and monomeric CRP. This method is based on the intracellular disulfide bonds, which are rapidly reduced in mCRP while pCRP is more resistant to reduction [35].

CRP as an acute phase protein is highly conserved in mammals and is thus also used as an inflammatory marker in veterinary medicine [36]. In pigs, CRP represents a typical acute phase protein and therefore has been well-characterized in different porcine cardiovascular and infectious models [37–39]. Moreover, pigs show one of the greatest similarities with humans with regard to physiology and the composition and function of the immune system [40]. Therefore, we assessed the impact of trauma on the CRP conformation using a porcine PT model. To better mimic the treatment of patients on the intensive care unit, PT pigs received the identical care including antibiotics, mechanical ventilation and fluid therapy [21]. Compared to sham animals, which also received similar care (anesthesia, intubation, ventilation), neutrophil invasion and activation was strongly increased in pPT.

In this porcine study, we found a time-dependent increase in plasma pCRP whereas mCRP was absent. Instead, we observed a conformation-specific pattern in the lungs as primary site of injury in this model. In the injured lung, we further found clear signs of neutrophil activity. Since CRP dissociation is described at the site of injury and is associated with aggravation of inflammation by interaction with immune cells [3], our data suggest a direct link between CRP and neutrophil activation. In vitro, we found that only mCRP, but not pCRP enhances chemotactic activity in human neutrophils*.* In this regard, mCRP as a strong opsonin [3] might be involved in debris clearance by recruiting phagocytic cells to the site of tissue damage and thus facilitating debris uptake after trauma. Moreover, our findings show mCRP at the site of inflammation, providing a novel pathophysiological mechanism for neutrophil activation based on mCRP-neutrophil interaction. Here, we demonstrate for the first time that mCRP induces sterile NET formation in vitro and ex vivo. We quantified NET formation microscopically through two distinct experimental approaches. By employing SYTOX staining and live-cell imaging, we visualized decondensed chromatin indicative of necrotic cells. In line, after cell fixation, citH3 staining as a marker of the early stages of NET formation colocalized with decondensed chromatin as occuring during NET formation. Both experiments demonstrated a significant induction by mCRP. Although an effective antimicrobial strategy, excessive NET formation at sites of inflammation can have pathophysiological consequences for the host. Under non-infectious conditions, NET formation has been shown to be involved in vascular injury, thrombosis and multiple organ injury due to the extensive release of bioactive enzymes including dsDNA, MPO, and elastase with detrimental effects on tissue homeostasis [41, 42].

As a potential mechanism for mCRP-induced NET formation, we suggest mCRP binding to a specific receptor on neutrophil surfaces. Supporting evidence came from several studies reporting Fcγ receptors (FcγR) as important receptors involved in CRP binding on leukocytes. While mCRP preferentially binds to FcγRI (CD64), native CRP is described to bind to FcγRII (CD32) on human neutrophils with the highest affinity [43]. Moreover, the mCRP-specific receptor CD64 is involved in respiratory burst and NET formation by an intracellular immunoreceptor tyrosine-based activation motif-mediated signaling system [44, 45]. Interestingly, CRP interaction with immune cells is also initiated via binding to lipid rafts rather than FcγRs on the cell surface [27, 46, 47], which might represent a possible FcγR-independent mechanism for mCRP-driven NET formation.

In vivo models of acute inflammation [3, 7, 13] showed that exposure of bioactive phospholipids in injured tissues including low density lipoprotein and lysophosphocholine might represent primary binding sites for pCRP and its dissociation to pCRP*/mCRP in trauma animals [3, 34]. Here, we provide evidence that the post-traumatic inflammatory microenvironment is a potent driver of CRP dissociation. Severe tissue injury is characterized by massive release of inflammatory mediators including ILs, and an early activation of the complement cascade [14]. We strongly suggest that the inflammatory mediators activate blood cells including platelets and leukocytes, which in turn expose an altered membrane phospholipid composition exposed by the action of phospholipase A_2_ and thus provide an ideal binding platform for pCRP with subsequent dissociation [13, 48–50]. Moreover, generation of mCRP within this inflammatory microenvironment also drives mCRP-induced NET formation ex vivo. This was further supported by blockade of NETosis in the presence of the small molecule inhibitor 1,6-bis-PC. 1,6-bis-PC is the only small molecule inhibitor for CRP and interacts with phosphocholine binding sites. Consequently, 1,6-bis-PC interaction abrogates binding of pCRP to known ligands such as low density lipoprotein and blocks CRP-mediated complement C1q activation [12, 51]. Thus, preventing CRP dissociation by 1,6-bis-PC represents a promising clinical approach for future interventions to modulate neutrophil activation. In rats with acute myocardial infarction, 1,6-bis-PC decreased infarcted tissue size and restrained cardiac dysfunction. In ischemic reperfusion injury, targeting CRP inhibited mCRP deposition in the kidney, leukocyte infiltration, and ROS generation [7]. Hence, blocking pCRP dissociation may represent a promising therapeutic approach in the prevention of direct and indirect organ injury. Moreover, neuroprotective features of targeting CRP may be beneficial in the setting of severe trauma, especially when including a traumatic brain injury [1, 12]. In addition, lung diseases may represent a worthwhile clinical setting for therapeutic intervention since studies demonstrated neutrophil-driven NET formation triggering acute respiratory distress syndrome and organ failure [25, 26]. In agreement, others found that CRP correlates with cell-free DNA, lung injury and disease severity in severe inflammatory conditions [52, 53].

Very recently, acetylcholine and nicotine have been found to have beneficial effects on reducing mCRP-induced inflammatory events [2, 54]. In our opinion, the effects of CRP conformational changes described herein are of relevance for a variety of clinical pathologies involving excessive local and/or systemic inflammation. These findings encourage further testing to determine whether our approaches prove suitable for clinical purposes.

## Conclusions

We have detected fundamental changes in neutrophil function induced by mCRP. Thus, future studies should also focus on the potential of the CRP conformation for immunomonitoring during inflammatory processes, presumably unveiling early signs of immune dysfunction before onset of organ damage and failure.

## Methods

### Clinical study in severely injured patients

A prospective clinical study was conducted in patients after severe PT (Injury Severity Score ≥ 32) that were admitted to the University Hospital Ulm between December 2013 and May 2015. The study protocol was approved by the Independent Local Ethics Committee of the University of Ulm (approval numbers 244/11 and 94/14). The study was registered on ClinicalTrials.gov, identifiers NCT00710411 and NCT02682550, and was performed in accordance with the Declaration of Helsinki and its recent modifications. Participants were recruited upon arrival at the Emergency department of Ulm University Medical Center and estimation of injury severity was performed by an emergency physician. Exclusion criteria were age < 18 years, pregnancy, infection with the human immunodeficiency virus, cardiogenic shock as the primary underlying disease, underlying hematologic disease, cytotoxic therapy given within the previous 6 months, and the presence of rapidly progressing underlying disease anticipating death within the next 24 h. Written informed consent was obtained from each subject.

### Plasma collection

Blood was drawn into serum, ethylenediaminetetraacetic acid (EDTA) and sodium citrate tubes and kept on ice. For CRP analysis using native western blot, EDTA blood was centrifuged at 2,200 × g for 15 min at 4 °C (*n* = 17, 0 h, 8 h, 24 h, 48 h, 120 h and 240 h). All supernatants were aliquoted on ice and stored at -80 °C until further analysis. Plasma CRP concentration was determined by the Clinical Chemistry at University Hospital Ulm using serum samples.

### Porcine PT study

The porcine PT study (pPT) was performed in accordance with the institutional guidelines and the German federal law regarding the protection of animals. The ethics proposal was approved by the Governmental Animal Care and Use Committee (“Landesamt für Natur, Umwelt und Verbraucherschutz Nordrhein-Westfalen,” Recklinghausen, Germany, AZ 81–02.04.2017. A412). All animals in the present study received human care according to the principles of the Guide for the Care and Use of Laboratory Animals (8th edition, NIH Publication, 2011, USA) and Directive 2010/63/EU on the protection of animals used for scientific purposes (Official Journal of the European Union, 2010). According to the 3R criteria by Russell and Burch [55], this study presents partial results obtained from a larger animal porcine multiple trauma model, which has been previously described in detail by Horst et al. [23] and modified as published by Guo et. al [21].

Sixteen pigs (German Landrace) weighing between 35 ±5 kg were included in the experiment after a 7 days period of acclimatization to the housing environment. All pigs were provided by a disease-free breeding facility and examined by a veterinarian upon delivery.

#### Instrumentation, anesthesia, and analgesia

Premedication was given with azaperone (4 mg/kg body weight) (Elanco, Greenfield, Indiana, USA) and intramuscular injection of ketanest (15 mg/kg body weight) (Pfizer, New York, USA).

General anesthesia was then maintained using Isoflurane (end-expiratory 1.2–2 vol.%) (AnaConDa®; Sedana Medical, Geretsried, Germany), fentanyl (40–90 µg/kg body weight/h) (Panpharma, Trittau, Germany), and remimazolam (0.4–2.0 mg/kg body weight/h) (PAION AG, Aachen, Germany). Fetanyl and remimozolam were given via a central venous line (V. jugularis dextra). For ventilation, a closed anesthesia device (EVITA IV; Dräger, Lübeck, Germany) was used in a volume-controlled mode under pressure limitation for P_insp_ of 30 cm H_2_O and lung-protective tidal volumes of 6–8 mL/kg body weight with target PaCO_2_ of 35–45 mmHg. A dialysis catheter was placed in the left femoral vein for later induction of hemorrhagic shock (HS). Furthermore, an arterial catheter (PiCCO; PULSION Medical Systems, Munich, Germany) was placed in the left femoral artery to monitor blood pressure (IntelliVue MP50; Philips, Germany). Furthermore, all animals received a suprapubic urine catheter (12.0 Fr, Cystofix®; Braun, Melsungen, Germany). After achieving stable baseline conditions(2 h after anesthesia induction), artificial warming by warm air blower and blankets (target body temperature 38 ± 0.5 °C) was stopped, O_2_ was defined at 21% for the following trauma period, simulating the ambient air, and trauma was induced.

#### Trauma induction

Trauma was induced by applying a blunt chest trauma with rib fractures and a tibia fracture. Moreover, a hemorrhagic shock was included drawing approximately 40% of the total blood volume and thus reaching a MAP at 40 ± 2 mm Hg. The HS was maintained for 90 min and was followed by a resuscitation phase by re-transfusion of the previously withdrawn blood as well as by additional fluids including Sterofundin ISO® (B. Braun, Melsungen, Germany) 0.5–2.0 mL/kg body weight/h and pediatric electrolyte solution 2.0 mL/kg body weight/h. Moreover, re-warming of the animals to 38.5 °C and increasing the O_2_ to 50% were done until the end of the experiment. Surgical care of the tibial fracture included was accomplished by an external fixator (Orthofix®) All posttraumatic treatments were performed according to recommendations in current guidelines.

#### Post-operative care

All post-operative procedures were similar to the intensive care protocol published study in [21]. Briefly, an intensive care period was initiated by administration of antibiotics (Ceftriaxon®, 2 g; repeated every 24 h). Moreover, to adapt the treatment of intensive care patients, and mechanical ventilation was supported by changing the animal’s position every 6–8 h from left or right side. Fluid therapy was performed by crystalloids including Pädiafusin II (Fresenius Kabi, Bad Homburg vor der Höhe, Germany) and Sterofundin ISO® (B. Braun), both 2 mL/kg body weight/h. Moreover, temperature of the animals was maintained within the physiological range via a warm air blower.

#### Sham group

Except of trauma and hemorrhagic shock, sham animals received the same type of instrumentation, anesthesia, and intensive care management with that in the trauma group. This also included a midline mini-laparotomy, which was performed for liver tissue sampling before trauma (liver sampling not relevant for this study).

#### Sampling

Organ sampling was performed after finalization of the experiment at the 72-h time point post trauma induction. In addition, due to the whole setting of the experiment, small liver tissue samples (0.5 cm × 0.5 cm) and blood samples were taken at each measurement time point and stored at –80 °C. Blood samplings were performed before trauma (0 h), after trauma (1.5 h), during resuscitation (2.5 h), after fracture stabilization (3.5 h), and at 5.5, 12, 24, 48, 67 and 72 h after trauma. Blood was drawn using EDTA monovettes (K3EDTA; SARSTEDT AG & Co, Germany). Plasma was obtained by blood centrifugation at 2,200 g for 15 min at 4 °C.

### CRP preparation

Human pCRP (236600, Merck Millipore, Darmstadt, Germany) was thoroughly dialyzed against Dulbecco’s PBS (DPBS) supplemented with 2 mM Ca^2+^. Structural and functional integrity of pCRP was verified by native gel electrophoresis. Commercial pCRP migrated at the same height as plasma CRP and showed no contaminations with mCRP. mCRP was generated by treating pCRP with 8 M urea in the presence of 10 mM EDTA for 2 h at 37 °C. mCRP was thoroughly dialyzed in low salt phosphate buffer (10 mM Na_2_HPO_4_, 10 mM NaH_2_PO_4_, and 15 mM NaCl, pH 7.4). To detect any bacterial contamination of CRP preparations, all reagents were tested for lipopolysaccharide contamination using a Limulus Amebocyte Lysate Chromogenic Endpoint Assay (HIT302, Hycult Biotech, The Netherlands). Endotoxin levels for pCRP and mCRP were below the detection limit (0.125 endotoxin units (EU)/ mL).

### Neutrophil isolation

Venous blood from healthy volunteers was drawn into tubes containing 3.2% sodium citrate from the antecubital vein (approved by the Local Independent Ethics Committee of the University of Ulm (number 459/18 and 462/18)). After mixing the blood with an equal volume of isotonic saline (0.9% NaCl), 20 mL was layered carefully over 10 mL of Ficoll-Paque (GE Healthcare, Freiburg, Germany) in a 50 mL centrifugation tube and cells were separated at 340 × *g* for 30 min with slow brakes. The supernatant containing plasma, mononuclear cells and Ficoll was removed, and the remaining pellet containing granulocytes and erythrocytes was mixed with dextran in 0.9% NaCl at a final concentration of 1%. After sedimentation of erythrocytes for 30 min, the supernatant was collected, and neutrophils were pelleted by centrifugation at 340 × *g* for 5 min. The remaining erythrocytes were lysed with distilled water, followed by addition of 2.7% NaCl to restore isotonic conditions. Neutrophils were pelleted and resuspended in HBSS^++^ medium at 5–10 × 10^6^/mL. HBSS^++^ supplemented with 0.1% BSA was used as assay medium for all in vitro experiments.

### Flow cytometry analysis for surface marker expression

For surface staining, freshly isolated neutrophils were resuspended in HBSS^++^ + 0.1% bovine serum albumin (BSA) at 5 × 10^6^/ mL. Neutrophil purity was 95 – 98% as assessed by light scatter characteristics in flow cytometry. After exposure to pCRP (50 µg/mL) and mCRP (50 µg/mL) for 60 min, cells were stained with mouse anti-human CD11b-APC (clone ICRF44, 301,309, biolegend) and rat anti-human CD62L-PE (clone MEL-14, 104,407, biolegend) or the isotype controls for CD11b (APC Mouse IgG1, κ Isotype Ctrl Antibody, MOPC-21, 400,119, biolegend) and CD62L (PE Rat IgG2a, κ Isotype Ctrl Antibody, clone RTK2758, biolegend) for 15 min at RT in the dark. Subsequently, cells were washed and fixed using 100 µl of freshly prepared cell fix (340,181, BD Biosciences, Heidelberg, Germany). Measurement of surface CD11b and CD62L was performed with at least 10,000 cells by flow cytometry and analyzed using FlowJo (Version 10.2, FlowJo, LLC, Ashland, OR, USA). Basic gating pattern based on FSC/SSC, doublet exclusion, removal of dead cells was performed. Based on surface marker expression, signal for respective expression molecules was selected using respective -isotype controls.

### Western blot analysis

After stimulation with pCRP or mCRP at 50 µg/mL for 1 h, PMNs were resuspended in cold RIPA buffer containing a protease- and phosphatase inhibitor cocktail. Cells were gently resuspended, sonified and kept on ice for 20 min. After a second sonification step, 4 × laemmli buffer including beta-mercaptoethanol was added and samples were denatured for 5 min at 95 °C. After electrophoresis, proteins were transferred on a PDVF membrane (GE Healthcare) and membranes were blocked in TBS + 1% Tween + 2% BSA (BSA/TBST) for 1 h. Subsequently, membranes were incubated with rabbit anti-human phospho-p38 MAPK (Thr180/Tyr182) antibody (4511, Cell Signalling Technology Inc, USA, 1:500 dilution in 5% BSA/TBST), rabbit anti-human phosphor-p47^phox^ (Ser345) antibody (PA5-37806, Invitrogen, Carlsbad, CA, USA, 1:500 dilution in 2% BSA/TBST), and rabbit anti-human MPO (79623, Cell Signalling Technology Inc, USA, 1:500 dilution in 5% milk/TBST) overnight at 4 °C. After washing, membranes were incubated with goat anti-rabbit-IgG-HRP secondary antibody (7074, Cell Signalling Technology Inc, USA, 1:1500 dilution in 5% BSA/TBST).

To assess the CRP conformation in plasma and ex vivo whole blood samples native western blot analysis was performed to prevent pCRP dissociation. For this purpose, samples were diluted in 2 × Laemmli buffer without the addition of beta-mercaptoethanol and without sample heating. Equal protein amounts in samples were then loaded on 10% gels (Bio-rad, fast). For electrophoresis, a native running buffer (Tris, Glycine,1/20 SDS) was used. Prior to blotting, proteins in gels were activated using Chemidoc XRS + and gels were boiled in running buffer containing 1% sodium dodecyl sulfate (SDS) and 1 mM dithiothreitol (DTT) for 5 min. After transfer to PVDF membranes, blocking was performed in 5% milk/TBST for 1 h. Membranes were incubated with mouse anti-human CRP (C1688, clone8, Sigma Aldrich, Steinheim, Germany, 1:500 in 5% milk/TBST) antibody and monoclonal mouse anti-human/pig CRP (clone 232026, R&D Minneapolis, MN, USA) overnight at 4 °C. After washing, membranes were incubated with horse anti-mouse-IgG-HRP secondary antibody (7076, Cell Signalling Technology Inc, USA, 1:5000 dilution in 5% milk/TBST) for 1 h at room temperature. Western clarity ECL solution (Biorad, Kidlington, UK) was used for signal development. A Chemidoc XRS + was used for detection of bands, and protein expression was normalized to total membrane protein using ImageLab software (Biorad; Kidlington, UK). Uncropped western blot images (Additional file 1: Fig. S5, S6).

### Ex vivo whole blood model

Following ethical approval by the Local Independent Ethics Committee of the University of Ulm (number 459/18) and after obtaining written informed consent, blood was drawn by peripheral venipuncture. The blood was collected in Lithium-heparin monovettes (Sarstedt, Nürnbrecht, Germany) Immediately after sampling, the blood was transferred carefully in a tubing system adapted and modified from Messerer et al. [56]. After addition of the inflammatory cocktail (Co), pCRP or a combination of Co + pCRP, the system was incubated for 120 min at 37 °C. For conditions with the CRP small molecule inhibitor, pCRP was pre-incubated with 1,6-bis-PC (1:100 molar ratio) 30 min at RT prior to the experiments. Following the incubation period, plasma was obtained by centrifugation at 340 × g for 5 min. The CRP conformation in plasma samples was assessed by native western blotting as described above. Moreover, PMNs were analyzed for surface marker expression of CD11b and CD62L by flow cytometry as described above. In addition, PMNs were isolated after incubation and cytospins were fixed in methanol for 15 min at RT. NET formation was assessed by citH3 and DNA (DAPI) staining.

### Immunostaining

For immunohistochemical staining, porcine lung and liver sections were deparaffinized in xylene, rehydrated in a descending alcohol series and boiled in sodium citrate buffer (3.0625 mg/mL, pH 6.0) for retrieval of antigen epitopes. Tissues were permeabilized using 0.1% Triton X-100 in TBS solution for 15 min. A blocking step was performed with 10% goat serum or donkey serum (depending on the secondary antibody) in TBS for one hour. After blocking, samples were incubated overnight in a humid chamber at 4 °C with the following antibodies: anti-human histone H3 citrulline (ab5103, Abcam, Cambridge, UK, 1:500 dilution in diluent (1% goat serum in TBS + 1% Tween 20)), anti-MPO (ab9535, Abcam, Cambridge, UK, 1:25 dilution in diluent (1% goat serum in TBS + 1% Tween 20)), conformation-specific mouse anti-human mCRP [57] (clone 9C9, used at a 1:500 dilution in diluent). Samples were washed, incubated with goat-anti-rabbit, goat-anti mouse IgG alkaline phosphatase (AP)-conjugated antibody (Jackson Immuno Reasearch Europe Ltd. Cambridgeshire, UK) or MPO Antibody (AF3667, R&D, Minneapolis, US, 1:200 dilution in diluent (1% donkey serum in TBS + 1% Tween 20) for 1 h at room temperature, followed by Dako REAL Detection System Chromogen Red (K5005, Agilent Santa Clara, CA, USA), and counterstained with Mayer´s hematoxylin (Sigma-Aldrich, Steinheim, Germany). Densitometric analysis of immunohistochemical mCRP staining was performed with Fiji-ImageJ or by ZEN 3.0 software.

To stain mCRP alone, 100 µl of 50 µg/ml mCRP was applied to a microscopic slide and allowed to dry. Afterwards, mCRP was fixed with 3.5% formaldehyde solution for 15 min at RT and boiled in sodium citrate buffer as described above. After blocking, mCRP staining was performed as described above.

PMN cytospins were centrifuged at 300 rpm for 2 min using a Shandon Cyto Spin 3 (Thermo Fisher Scientific, Waltham, MA, USA) and subsequently fixed using a 3.5—3.7% formaldehyde solution for 15 min at RT. After three washing steps in TBS, slides were blocked with 10% goat or donkey serum in TBS for 1 h.

For immunofluorescence, the sections or slides were treated as described above. As secondary antibodies, AlexaFluor486®-conjugated goat anti-rabbit IgG antibody (Invitrogen Carlsbad, CA, USA), AlexaFluor568®-conjugated goat anti-rabbit IgG (Invitrogen Carlsbad, CA, USA), AlexaFluor568®-conjugate goat α-mouse IgG (Jackson Immuno Reasearch Europe Ltd. Cambridgeshire, UK), AlexaFluor^TM^568-conjugate donkey anti-goat IgG (Invitrogen Carlsbad, CA, USA) or AlexaFluor^TM^488-conjugate donkey anti-rabbit IgG (Invitrogen Carlsbad, CA, USA) were used at a dilution of 1:500 and incubated for 1 h, respectively. After washing, the specimens were directly covered with Prolong™ Gold antifade reagent with DAPI (P36931, Invitrogen, Carlsbad, CA, USA) for counterstaining of nuclei.

PMNs on cytospins were assessed counting the proportion of NETting cells, i.e. with decondensed chromatin or positive for citH3, in a blinded fashion and normalizing to the unstimulated control.

### Reactive oxygen species detection

Isolated PMNs were adjusted to 5 × 10^6^ cells/mL in HBSS^++^ and were incubated with Dihydrorhodamine (DHR) 123 (sc-208306, Santa Cruz Biotechnology, Heidelberg, Germany 1:1000 dilution) at 37 °C for 30 min protected from light. After incubation, cells were washed and resuspended in HBSS^++^ containing 0.1% BSA. PMNs were seeded on 96-well plates and incubated for 10 min at 37 °C. Baseline fluorescence was determined with a fluorescence reader. PMNs were stimulated with 50 µg/mL pCRP, or 50 µg/mL mCRP and incubated at 37 °C protected from light. Measurements were performed directly, 10 min, 30 min, 60 min, and 120 min after incubation FluoroSkan Ascent® cytofluorometry (excitation filter 485 nm/emission filter 538 nm).

### NET formation in neutrophils as detected by live-cell imaging

After neutrophil isolation from five healthy volunteers, 5 × 10^6^ cells/ml were resuspended in HBSS^++^ buffer supplemented with 0.1% BSA. PMNs were stained with a final concentration of 10 µg/mL Hoechst 34,580 (Sigma-Aldrich, Steinheim, Germany) and 1 µM SYTOX AADvanced death cell stain (Invitrogen Carlsbad, CA, USA). To induce NET formation, cells were treated with either 100 nM PMA, 50 µg/ml pCRP, 50 µg/ml mCRP, or HBSS^++^ buffer supplemented with 0.1% BSA as control. Cells were imaged using the Live-Cell-Image Fluorescence microscope Leica DMI6000 B with incubation-cam where cells were incubated at 37 °C and 5% CO_2_ up to 4.5 h. Cells positive for SYTOX as a fraction of at least 200 total cells after incubation for 1.5 h were counted in a blinded fashion in 200 × magnification.

### NET formation markers in neutrophil supernatants

After neutrophil isolation and exposure to pCRP and mCRP for 1 h, supernatants were collected by centrifugation at 340 × g for 5 min. To exclude remaining cells, a second centrifugation at 16 000 × g for 2 min was performed. Cell-free supernatants were collected and immediately analyzed for extracellular DNA content using a dsDNA assay kit (Qubit® dsDNA HS Assay Kit, Invitrogen Carlsbad, CA, USA) according to the manufacturer’s instructions. Elastase concentrations in neutrophil supernatants were determined using a commercially available enzyme-linked immunosorbent assay (ELISA) kit strictly in accordance with the manufacturer’s protocol (DY9167-05, R&D, Wiesbaden-Nordenstadt, Germany).

### Myeloperoxidase assay

For assessment of myeloperoxidase (MPO) activity, neutrophil supernatant or standard human MPO (475,911, Merck, Darmstadt, Germany) was incubated with 100 μg/mL tetramethylbenzidine (860,336, Merck, Darmstadt, Germany) and H_2_O_2_ (0.0016%) at 37 °C in a 96 well plate. After 5 min, the reaction was terminated by the addition of 2 M H_2_SO_4_, and the absorbance at 450 nm was determined using a spectrophotometric reader (Tecan Sunrise, Thermo).

### Chemotaxis

Isolated neutrophils (5 × 10^6^ cells/mL in DPBS) were labeled with 2′,7′-bis-(2-carboxyethyl)-5-(and -6)-carboxyfluorescein (Abcam, Cambridge, UK). Following a washing step with DPBS, cells were pre-incubated with pCRP (50 µg/mL) or mCRP (50 µg/mL) for 30 min at 37 °C. The neutrophils were loaded into the upper chambers of a 96-well chemotaxis mini-chamber. The lower chamber was loaded with N-formylmethionyl-leucyl-phenylalanine (100 nM) and separated from the upper chamber by a polycarbonate membrane of 3-μm porosity. The cells were incubated in the mini-chamber (1C96, Neuro Probe, USA) for 30 min at 37 °C. To determine the neutrophil chemotactic activity, the number of cells that had migrated through the polycarbonate filters to the lower surface was determined by FluoroSkan Ascent® cytofluorometry (excitation filter 485 nm/emission filter 538 nm). Samples were assayed at least in quadruplicates.

### Phagocytosis assay

Venous whole blood containing heparin as anticoagulant was incubated with pCRP or mCRP for 30 min. A commercial phagocytosis assay was performed according to the manufacturer’s instructions. Briefly, *Escherichia coli* bioparticles labeled with fluorescent pH-sensitive pHrodo® Red Succinimidyl Ester (A10025, Thermo Fisher Scientific, Waltham, United States) were added to the whole blood samples and incubated at 37 °C. After 15 min, erythrocytes were lysed, cells were fixed and flow cytometry analysis was performed. The pHrodo® dye has a low fluorescence intensity at neutral pH, but emits a several-fold higher fluorescence upon acidification in the lysosome, facilitating the specific detection of phagocytosed particles [58, 59]. PMNs were gated based on forward scatter (FSC)/side scatter (SSC) properties. For quantification of phagocytosis in acidic endosomes, PE mean fluorescence intensity (MFI) was evaluated.

### Statistical analysis

Every experiment consists of at least 3 independent biological replicates to allow for statistical analysis. No data were excluded from analyses. Experimental results were compared using Student´s t-test for two groups or one-way analysis of variance (ANOVA) followed by Student–Newman–Keuls post-hoc or with Dunn´s test for three or more groups. Correlation analyses were performed using Pearson Product Moment Correlation. For all ANOVA testing, no formal statistical test on normality was applied due to its limited validity regarding the available sample size. Results are presented as mean ± standard error of the mean. A *p*-value < 0.05 was considered statistically significant. All statistical analyses were performed using SigmaPlot (Version 14.0, Systat Software, Erkrath, Germany).

## Supplementary Information

Additional file 1: Fig. S1. Correlation analysis of plasma CRP with clinical parameters after huPT. Fig. S2. Conformation-specific CRP histological staining in porcine organ sections. Fig. S3. Apoptosis and NETosis in stimulated neutrophils. Fig. S4. Influence of the CRP conformation on distinct neutrophil functions. Fig. S5. Uncropped western blot membranes for Figs. 1 and 2. Fig. S6. Uncropped western blot membranes for Figs. 2 and 3.

## Data Availability

All data generated or analyzed during this study are included in this published article and its additional files. No new materials were generated in this study.

## Abbreviations

ANOVA: Analysis of variance
AP: Alkaline phosphatase
BSA: Bovine serum albumin
citH3: Citrullinated histone 3
Co: Inflammatory cocktail
CRP: C-reactive protein
dsDNA: Double-stranded DNA
DTT: Dithiothreitol
EDTA: Ethylenediaminetetraacetic acid
FcγR: Fcγ receptors
FSC: Forward scatter
HBSS: Hank's Balanced Salt Solution
HS: Hemorrhagic shock
huPT: Human polytrauma
IL: Interleukin
MAPK: Mitogen-activated protein kinase
mCRP: Monomeric CRP
MFI: Mean fluorescence intensity
MPO: Myeloperoxidase
NADPH: Nicotinamide adenine dinucleotide phosphate
NET: Neutrophil extracellular trap
PAD4: Peptidylarginine deiminase 4
pCRP: Pentameric CRP
PMA: Phorbol-12-myristat-13-acetate
pPT: Porcine polytrauma
PT: Polytrauma
ROS: Reactive oxygen species
SDS: Sodium dodecyl sulfate
SSC: Side scatter
