# Supplementary material for: A conformational change of C-reactive protein drives neutrophil extracellular trap formation in inflammation
Source: BMC Biol. 2025 Jan 7;23:4. doi: 10.1186/s12915-024-02093-8 (PMC11708171; doi:10.1186/s12915-024-02093-8)
Supplement: Supplementary file 1 — Additional file 1: Fig. S1. Correlation analysis of plasma CRP with clinical parameters after huPT. Fig. S2. Conformation-specific CRP histological staining in porcine organ sections. Fig. S3. Apoptosis and NETosis in stimulated neutrophils. Fig. S4. Influence of the CRP conformation on distinct neutrophil functions. Fig. S5. Uncropped western blot membranes for Figs. 1 and 2. Fig. S6. Uncropped western blot membranes for Figs. 2 and 3. [file 12915_2024_2093_MOESM1_ESM.pdf]

Supplementary Figure S1

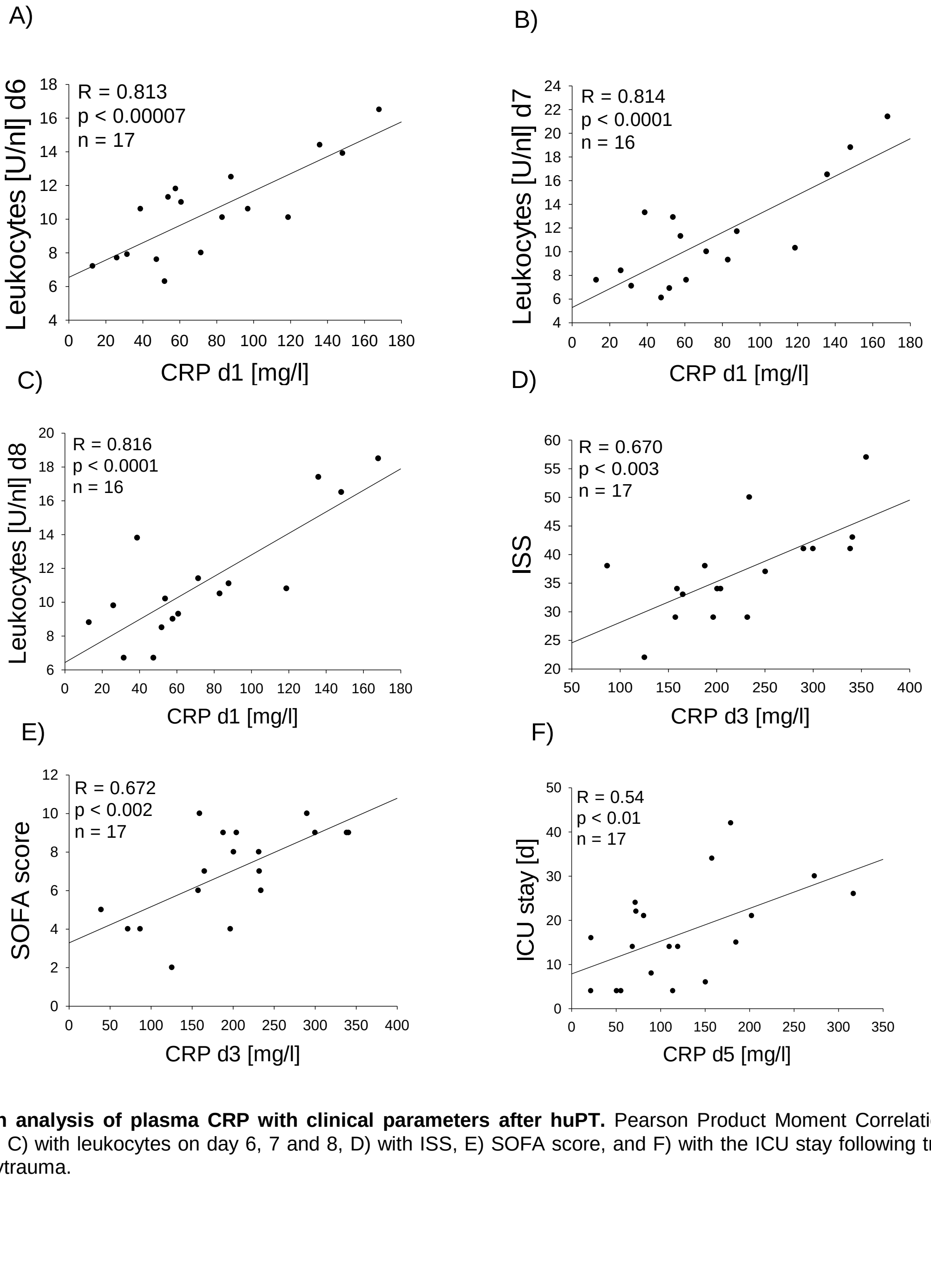

Correlation analysis of plasma CRP with clinical parameters after huPT. Pearson Product Moment Correlation of plasma CRP (A, B, C) with leukocytes on day 6, 7 and 8, D) with ISS, E) SOFA score, and F) with the ICU stay following trauma. huPT, human polytrauma.

Supplementary Figure S2

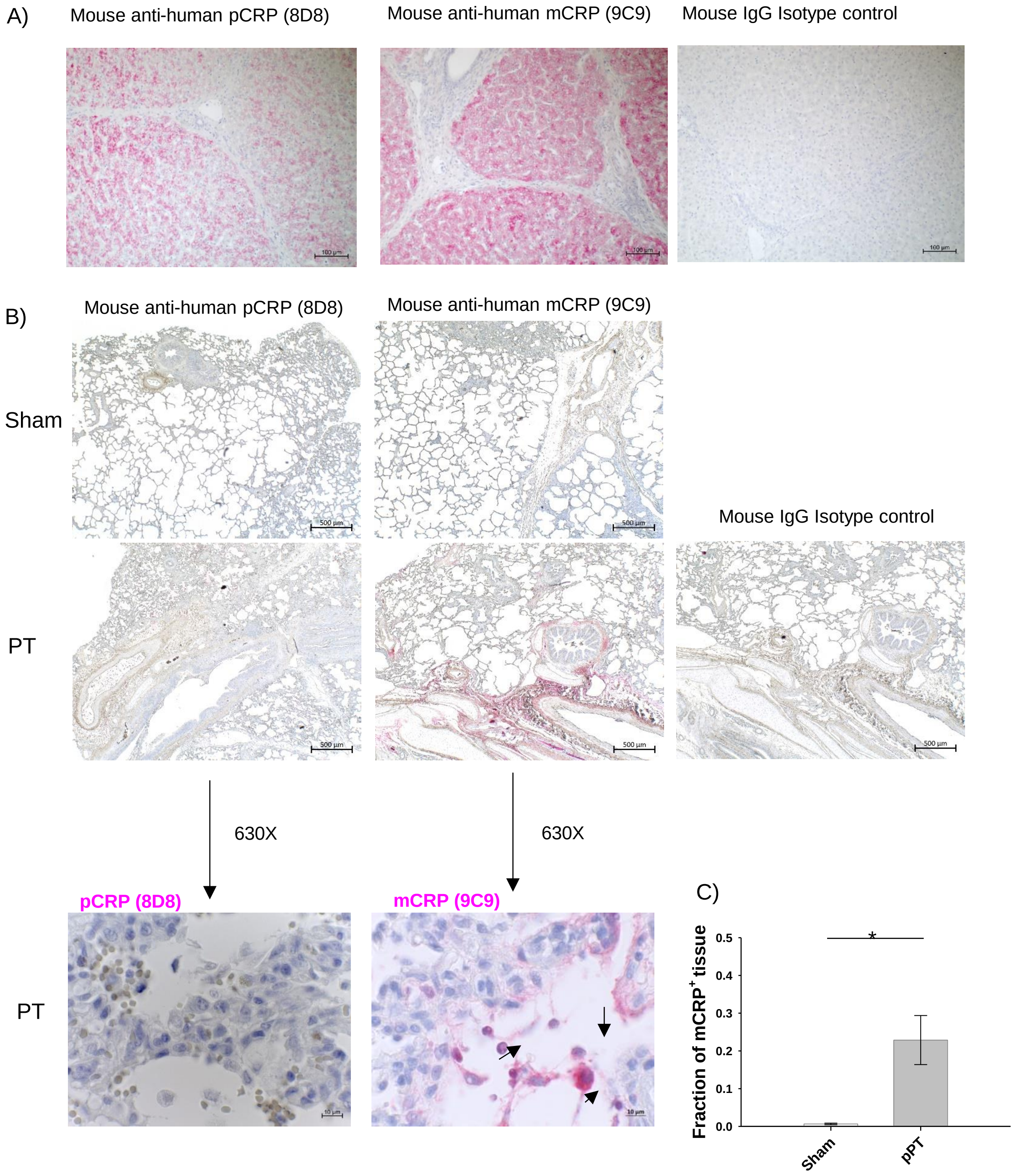

Conformation-specific CRP histological staining in porcine organ sections. A) Representative immunohistochemistry images of porcine liver sections using mouse anti-human pCRP (clone 8D8, left panel) and mouse anti-human mCRP (clone 9C9, middle panel) shows human and pig species cross-reactivity. B) Immunohistochemistry of pig lung section of sham-operated and pPT animals with CRP conformation-specific antibodies at 25x magnification. Lower panel depicts images with 630x magnification. Mouse IgG k antibody was used for specificity control (right panel). Alkaline phosphatase conjugated secondary goat anti-mouse antibody was used and developed using DAKO real detection kit. C) Densitometric analysis of lung tissues stained for mCRP. The fraction of mCRP-positive areas of the total tissue was analysed for n=3 sham and n=7 pPT lung tissue sections.

Supplementary Figure S3

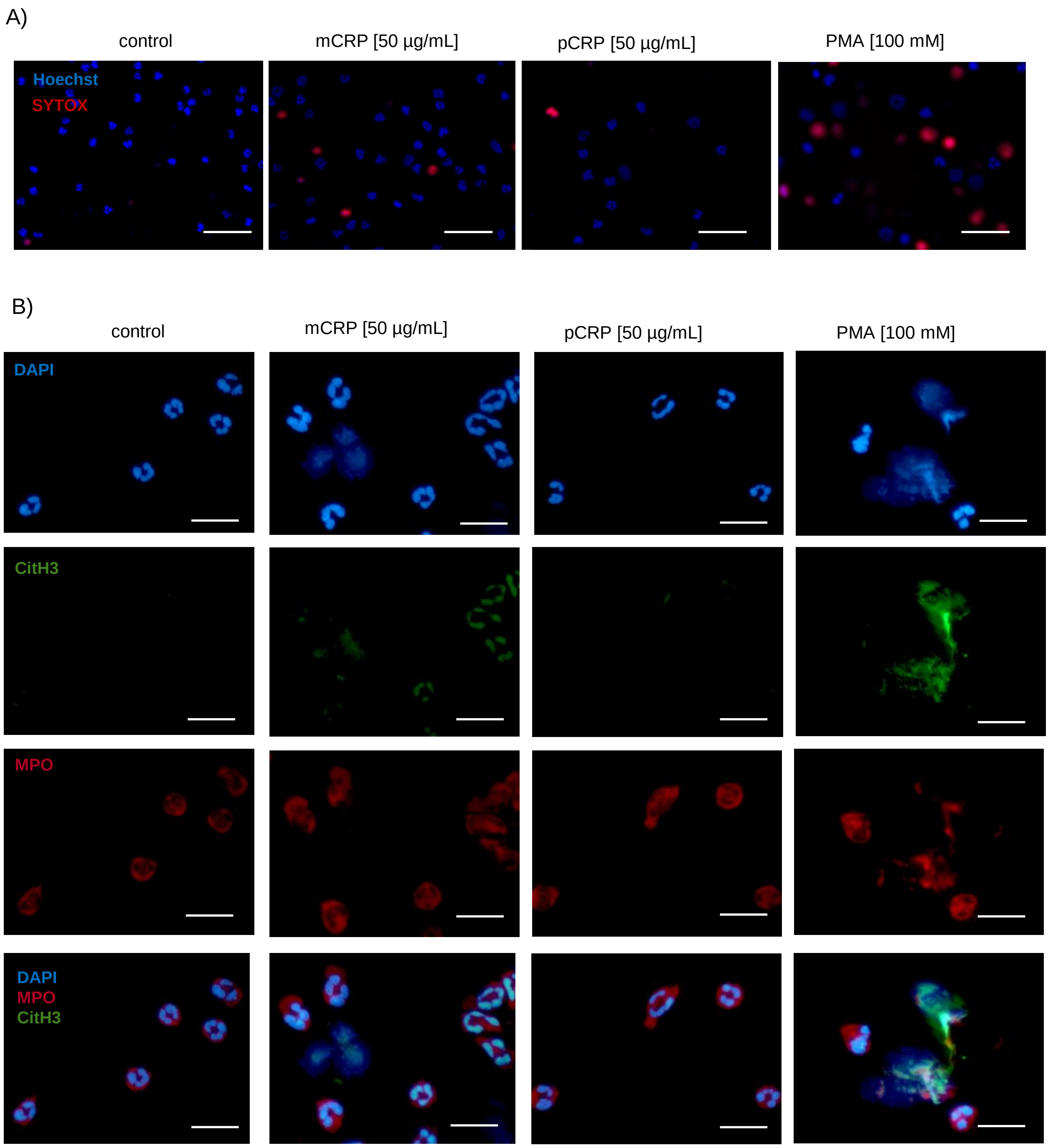

Apoptosis and NETosis in stimulated neutrophils. A) Immunofluorescence image of isolated PMNs after treatment with pCRP (50 µg/mL), mCRP (50 µg/mL), and PMA (100 mM) for 1.5 h. DNA was stained using SYTOX (red) and Hoechst (blue), scale bar indicates 25 µm. **B)** Immunofluorescence image of PMN cytospin after treatment with pCRP (50 µg/mL), mCRP (50 µg/mL), and PMA (100 mM) for 1 h. Images depicting individual fluorescence channels displaying MPO (red), citH3 (green), and DAPI (blue), scale bars indicate 25 µm.

Supplementary Figure S4

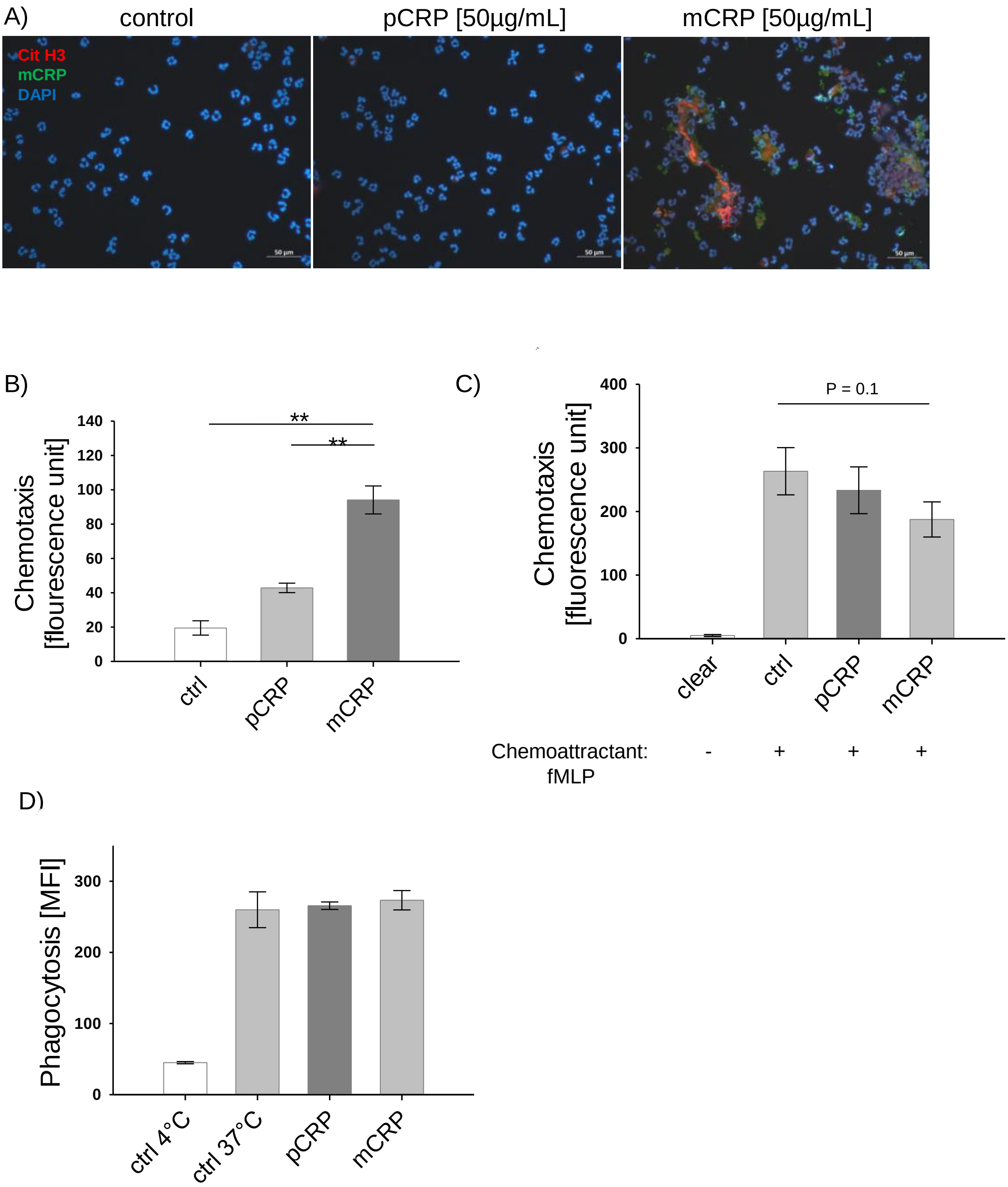

Influence of the CRP conformation on distinct neutrophil functions. A) Immunofluorescence images of citH3 (red), mCRP (green) and nuclei (DAPI) staining of fixed neutrophils after exposure to pCRP (50µg/mL) and mCRP (50µg/mL) for 60 min. B) Chemotaxis activity of neutrophils using a Boyden chamber assay. pCRP and mCRP were used as chemoattractants. $HBSS^{++}$ buffer served as control. n=4 per group C) Chemotaxis activity of pCRP- and mCRP- incubated neutrophils to the chemoattractant N-formyl-L-methionyl-L-leucyl-phenylalanine (fMLP). As control, a clear condition with buffer was used as chemoattractant, n=4 per group. D) Phagocytosis of E. coli bioparticles was assessed by fluorescence intensity using flow cytometry analysis. After incubation with pCRP and mCRP for 60min, E. coli bioparticles were included for 15 min at 37°C. As baseline control, non-stimulated cells were used. For negative control, cells were incubated on ice, n=3.

Supplementary Figure S5

A)

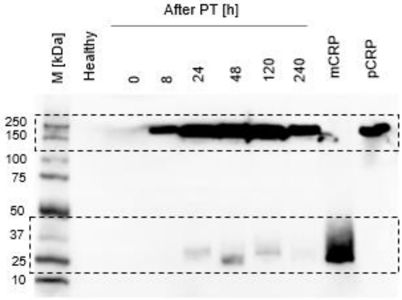

B)

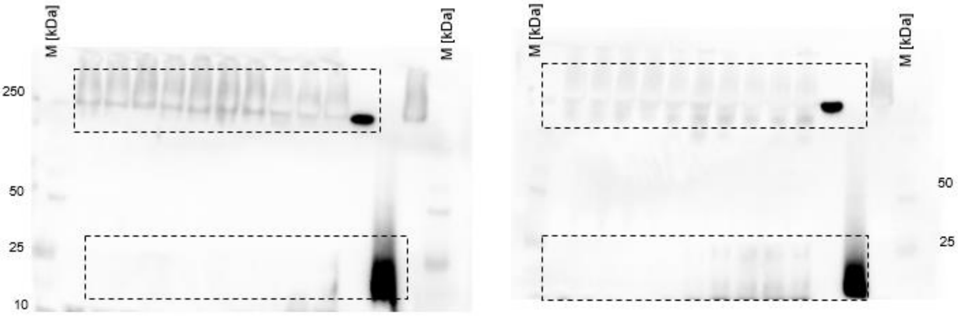

C)

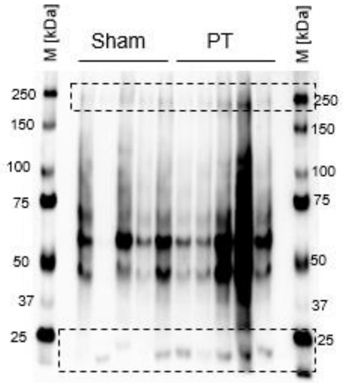

D)

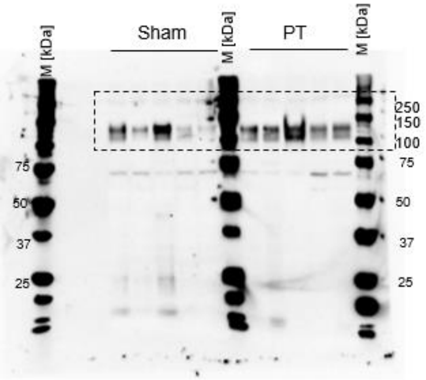

E)

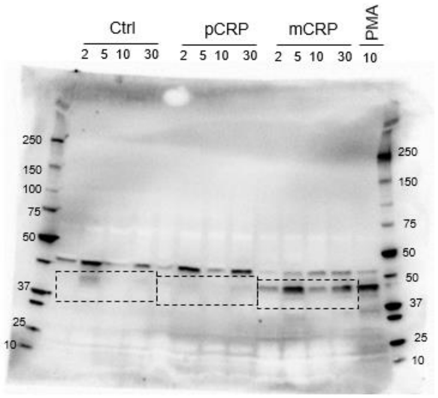

Uncropped western blot membranes. A) Full unedited membrane for Figure 1B: Representative native CRP immunoblot including healthy and huPT plasma samples. B) Full unedited membrane for Figure 1D: Representative immunoblots of porcine plasma samples obtained from sham and pPT animals at different sampling times and probed for pCRP and mCRP. C) Full unedited membrane for Figure 1E and 1F: pCRP and mCRP in porcine lung tissue lysates. D) Full unedited membrane for Figure 1G: Immunoblot probed for MPO in porcine lung tissue lysates. E) Full unedited membrane for Figure 2F (upper panel). Representative immunoblot for Ⓟ-p38 MAPK after incubation with pCRP and mCRP.

Supplementary Figure S6

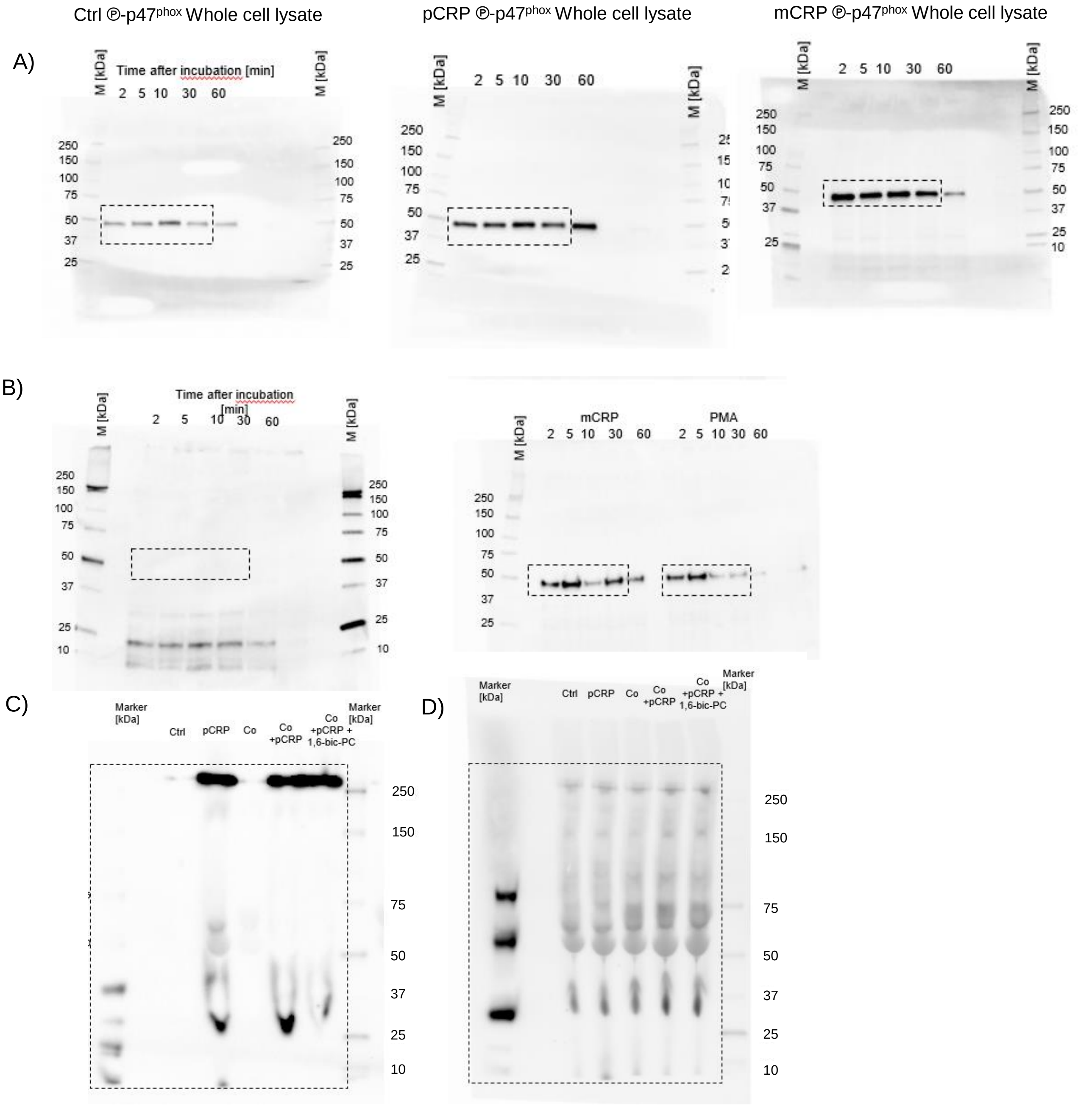

Uncropped western blot membranes for Figs. 2 and 3. A) Full unedited membrane for Figure 2F (middle panel): Representative immunoblots for ®-p47phox in PMN cell lysates after incubation with pCRP and mCRP. B) Full unedited membrane for Figure 2F (lower panel): Representative immunoblots for Membrane-associated ®-p47phox in PMN cell lysates after incubation with pCRP and mCRP. C) Full unedited membrane for Figure 3C: Native western blot image probed for CRP conformation after human ex vivo whole blood stimulation with a clinically relevant inflammatory cocktail alone or in combination with pCRP or pCRP + 1,6-bis-PC. D) Stain-free total protein blot image for the normalization of western blot image shown in Figure 3C.
